# Confronting Cholelithiasis: A Case Series of Patients With Sickle Cell Disease and Gallstones

**DOI:** 10.7759/cureus.80558

**Published:** 2025-03-14

**Authors:** Javed Jagroo, Omar A Oudit, Corey Knowles, Srikanth Adidam Venkata

**Affiliations:** 1 Internal Medicine, Brookdale University Hospital Medical Center, Brooklyn, USA; 2 Neurology, Downstate Neurology at One Brooklyn Health, Brooklyn, USA

**Keywords:** cholelithiasis, endoscopic retrograde cholangiopancreatography (ercp), gallbladder diseases and gallstones, hemolysis, hepatobiliary complications, sickle cell disease complications

## Abstract

Sickle cell disease (SCD) is a prevalent hemoglobinopathy that leads to various complications, including hepatobiliary diseases, which are often underrecognized. Cholelithiasis and choledocholithiasis, resulting from accelerated pigment gallstone formation due to increased hemolysis, are common in SCD patients. This case series aims to raise awareness of the heightened risk of biliary disease in SCD and emphasize the need for early screening and management strategies.

We present four cases of adult SCD patients who developed gallstones, each requiring medical intervention for biliary complications. The first case involved a 47-year-old woman with SCD and choledocholithiasis, presenting with right upper quadrant pain and jaundice. She underwent a laparoscopic cholecystectomy and endoscopic retrograde cholangiopancreatography (ERCP) for stone removal. The second case described a 25-year-old woman with a history of SCD, who had hyperbilirubinemia and dilated common hepatic duct. She successfully underwent ERCP and laparoscopic cholecystectomy after cholelithiasis was confirmed. The third case involved a 34-year-old man with acute abdominal pain and right-sided testicular pain, diagnosed with gallstones and early cholecystitis. Despite plans for surgery, he signed out against medical advice. The fourth case was a 28-year-old woman with recurrent acute chest syndrome, who presented with abdominal pain and was found to have multiple gallstones. She underwent laparoscopic cholecystectomy, but developed acute chest syndrome postoperatively, requiring exchange transfusion. All patients experienced post-surgical recovery, although one patient had complications related to acute chest syndrome.

Hepatobiliary manifestations in SCD include cholelithiasis, choledocholithiasis, and other liver-related issues. The formation of pigmented gallstones is a direct result of chronic hemolysis, where increased bilirubin levels contribute to stone formation. The incidence of cholelithiasis in SCD patients is significant, with factors such as hemoglobin levels and bilirubin elevation increasing the likelihood of gallstone formation. Symptomatic gallstones often present with right upper quadrant pain, which can be exacerbated by vaso-occlusive crises. Early intervention, including elective cholecystectomy, can help prevent complications like choledocholithiasis, biliary colic, and cholecystitis. Despite a lack of formal guidelines for routine cholecystectomy in asymptomatic patients, evidence supports early surgical management to prevent further complications and reduce the need for emergent procedures.

This case series highlights the importance of early screening and elective cholecystectomy for SCD patients at risk of biliary disease. Early intervention can prevent acute complications, reduce hospitalizations, and improve the quality of life for patients with SCD. Future research and guidelines should focus on establishing protocols for screening and management of gallstone-related hepatobiliary complications in this population.

## Introduction

Sickle cell disease (SCD) is one of the most common hemoglobinopathies in the world, affecting approximately 100,000 Americans, with SCD occurring in one in 365 black American births. The disease is associated with multiple health complications that have been well documented, and the American Society of Hematology has issued guidelines addressing these [1]. There continues to be research and reviewing of data aimed at delivering screening and treatment guidelines for SCD related cardiopulmonary disease, renal disease, pain management, cerebrovascular disease, blood transfusion support, and stem cell transplantation [1]. However, there is a notable deficiency in such guidelines for SCD-related hepatobiliary diseases. This may be attributed to diagnostic dilemmas and greater clinical focus on the more common, well known SCD related complications.

Patients with sickle cell disease characteristically develop episodes of intravascular hemolysis that clinically present with fatigue, malaise, pallor, and if severe enough can lead to more serious symptoms such as chest pain and hemodynamic instability. The laboratory markers that indicate intravascular hemolysis include decreased hemoglobin (Hgb), increased indirect bilirubin, increased lactate dehydrogenase (LDH) and haptoglobin. This hemolysis contributes to the development of calcium bilirubinate stones at a much greater rate than non-SCD individuals. Thus, this pathology accelerates the development of comorbid biliary involvement and raises the question of best management as it relates to this hepatobiliary disease in these select patients.

The purpose of this study is to increase physician awareness of the accelerated development of cholelithiasis and choledocholithiasis that occur in SCD patients in consequence of enhanced pigment gallstone formation. We present to you a case series of patients with SCD who all developed gallstones. Their hospital courses were all confounded by complications of cholelithiasis requiring various interventions.

## Case presentation

Case 1 involves a 47-year-old woman with SCD and cholelithiasis who presented with generalized body pain characteristic of crisis pain. She attributed her symptoms to the changing climate during fall but noted decreased appetite due to postprandial abdominal pain for a week. Examination revealed right upper quadrant (RUQ) abdominal pain and icterus. On admission her Hgb was found to be 6.8 g/dL (baseline 6.5-7.5 g/dL) with elevated indices of hemolysis. Reticulocyte count was 8.47%, direct bilirubin 0.93 mg/dl, total bilirubin 4.5 mg/dl, LDH 1542 U/L, white blood cell (WBC) 13.4 X 10^3^/uL (Table 1).

**Table 1:** Laboratory results of each patient on admission.

Laboratory marker	Reference range	Case 1	Case 2	Case 3	Case 4
Hemoglobin	11.4- 15.5 g/dL	6.8 g/dL	9.3 g/dL	8.7 g/Dl	8.9 g/dL
White blood cell count	4.5 -10.2 x 10^3^/Ul	13.4 x 10^3^/uL	12.3 x 10^3^/uL	25.0 x 10^3^/Ul	10.7 x 10^3^/Ul
Reticulocyte count %	0.5-1.5%	8.47%	15.35%	3.15%	9.21%
Total bilirubin	0.3-1.0 mg/dL	4.2 mg/dL	7.5 mg/dL	1.9 mg/dL	3.2 mg/dL
Direct bilirubin	0.03-0.18 mg/dL	0.93 mg/dL	0.84 mg/dL	0.41 mg/dL	0.53 mg/dL
Lactate dehydrogenase	140-271 U/L	1542 U/L	439 U/L	260 U/L	393 U/L
Alkaline phosphatase	34-104 U/L	211 U/L	88 U/L	55 U/L	67 U/L

**Figure 1:**
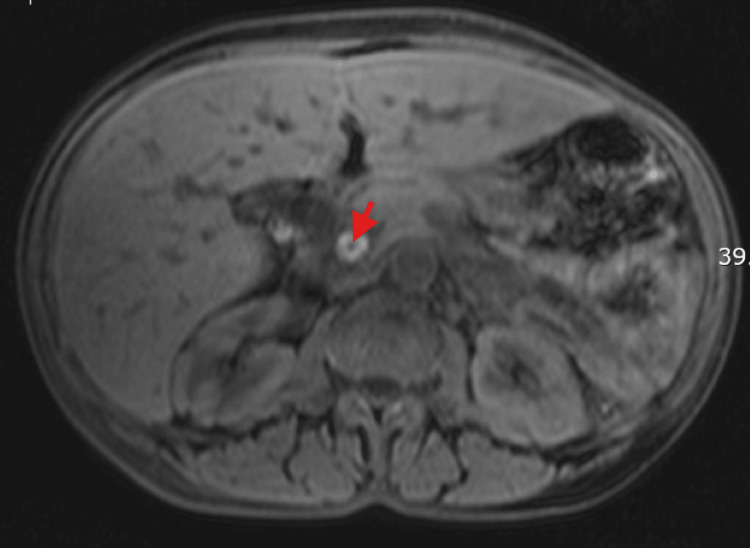
A magnetic resonance cholangiopancreatography image demonstrating a calculi (red arrow) within the common bile duct which is also dilated (case 1).

Abdominal imaging with magnetic resonance cholangiopancreatography (MRCP) conveyed choledocholithiasis with common bile duct dilation of 1.9 cm but no features to suggest cholangitis or pancreatitis (Figure 1). A cholecystectomy and subsequent endoscopic retrograde pancreatography (ERCP) was performed, including sphincterotomy, stone extraction, and stent placement due to residual obstruction with no complications. She then underwent laparoscopic cholecystectomy with successful recovery post procedure.

**Figure 2:**
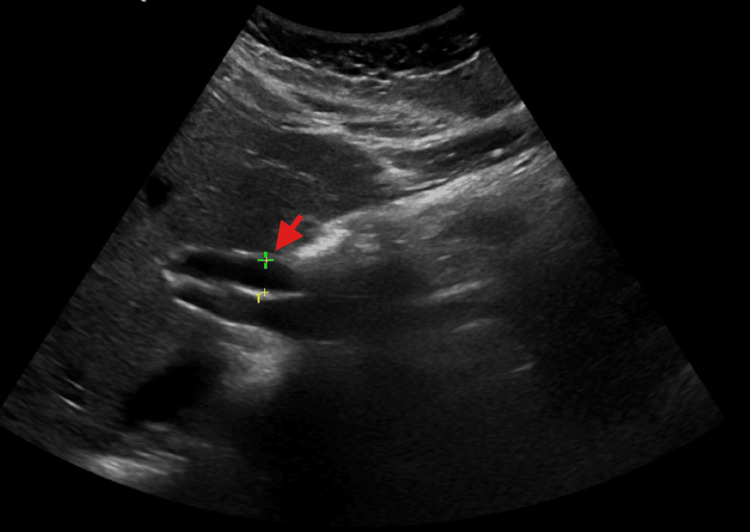
Ultrasound image of abdomen demonstrating dilated common bile duct (red arrow) of 7.2 mm (case 2).

Case 2 highlights a 25-year-old woman, originally from Haiti, presenting with a right medial malleolar ulcer which appeared one week prior. On admission she was found to have a Hgb of 9.3 g/dL, total bilirubin of 7.5 mg/dl, and LDH of 439 U/L. She revealed that both her parents were sickle cell trait positive. Her sickle cell screen was positive, peripheral smear revealed sickled cells and hemoglobin S fraction was 87.8%. Clinically she was not in vaso-occlusive crisis and an abdominal sonogram was done to further investigate her isolated hyperbilirubinemia, revealing cholelithiasis and dilated common bile duct of 7.2 mm (Figure 2).

**Figure 3:**
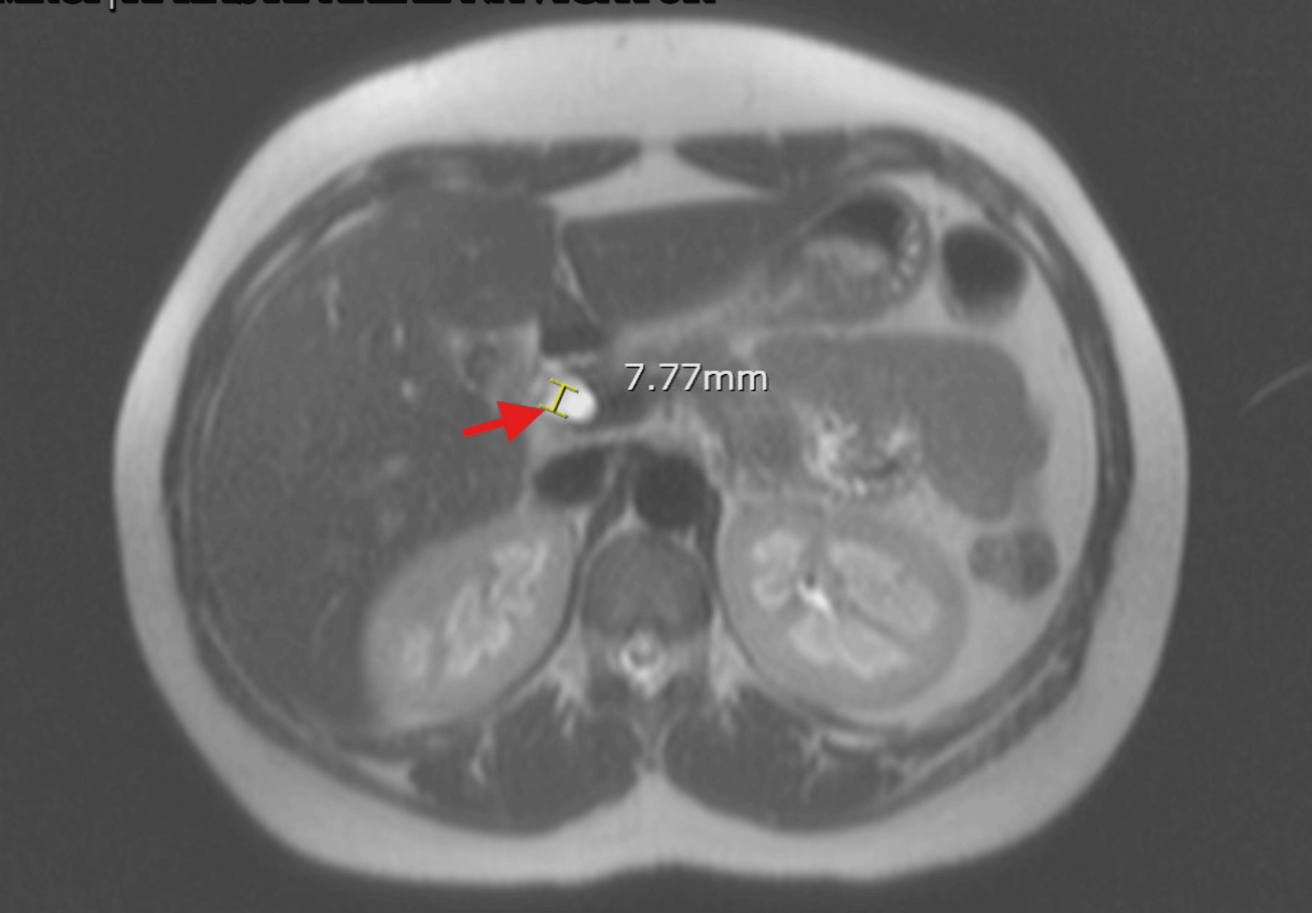
Axial view of magnetic resonance cholangiopancreatography of case 2 which revealed a distal 4 mm common bile duct stone and dilated common bile duct of 7.77 mm (red arrow).

MRCP confirmed cholelithiasis and choledocholithiasis (Figure 3). She remained clinically stable and ERCP was performed with two pigmented stones removed from the bile duct. She subsequently underwent laparoscopic cholecystectomy and recovered successfully. Her Hgb remained stable and bilirubin levels normalized post intervention.

**Figure 4:**
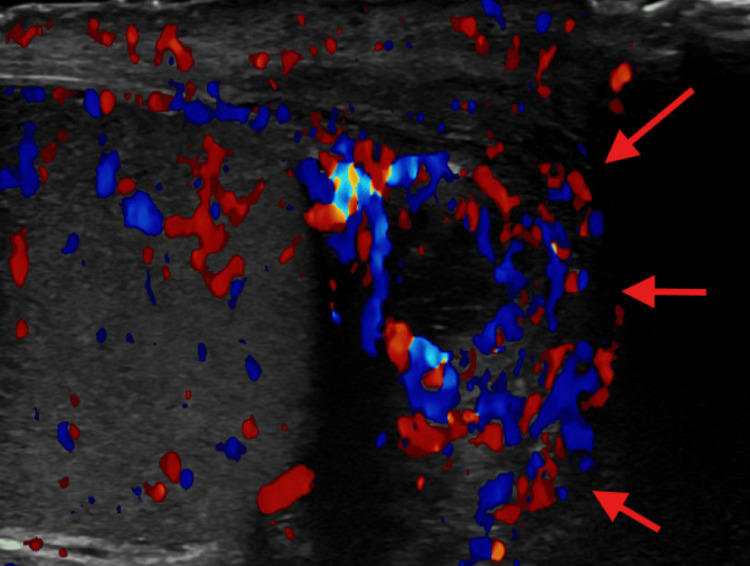
Doppler ultrasound in case 3 of right testicle and epididymal tail which is thickened with mild hyperemia (red arrows).

Case 3 describes a 34-year-old man with SCD who presented with a four-day history of right-sided testicular pain and generalized joint pain. Initial lab work revealed Hgb of 8.7 g/dL (baseline 8-9 g/dL), WBC 25 X 10^3^ /uL with neutrophil 78%, reticulocyte count 3.15%, and LDH 260 U/L. Scrotal ultrasound indicated thickening of the right epididymal tail associated with mild hyperemia suggesting acute right epididymitis (Figure 4).

**Figure 5:**
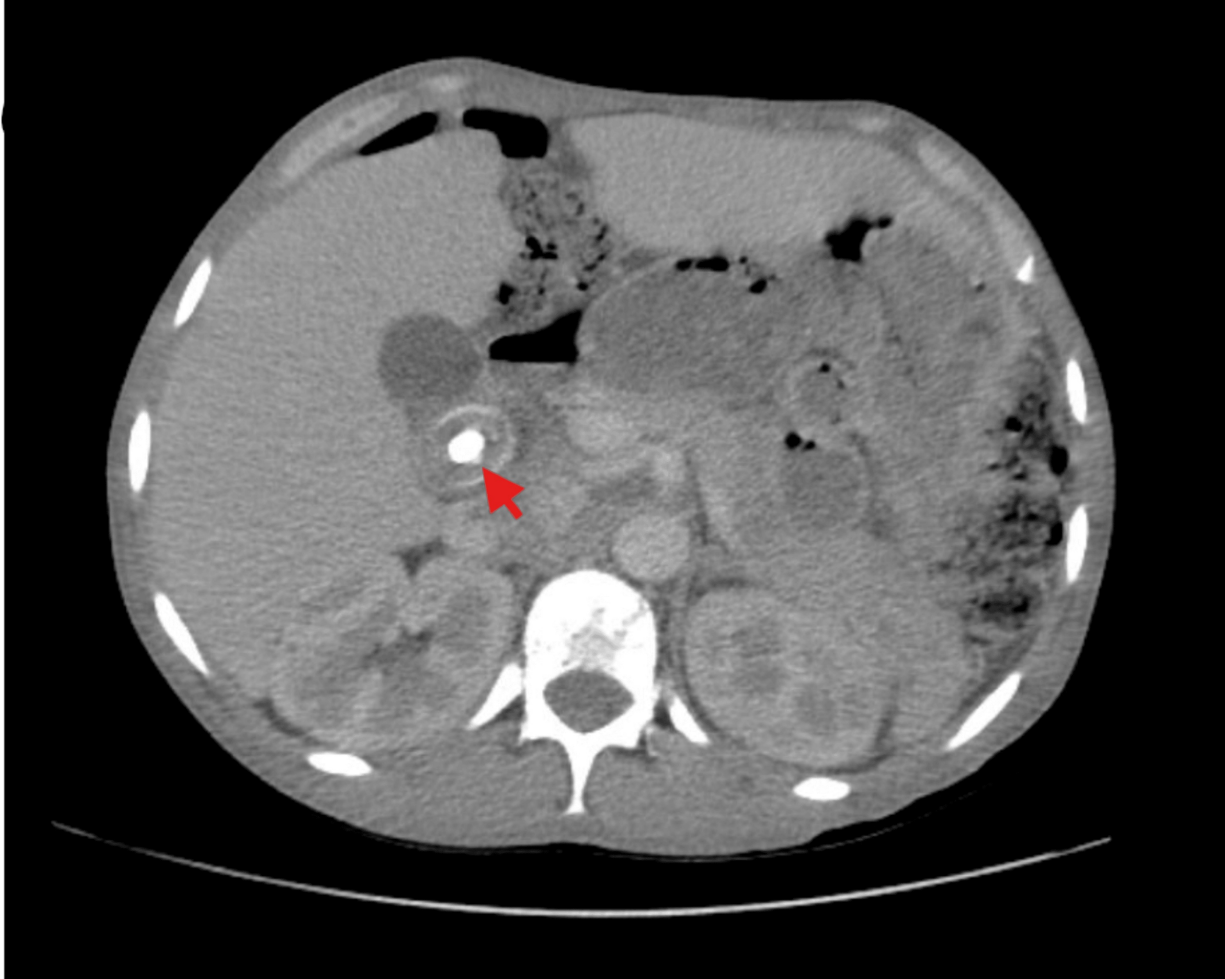
Axial view of computed tomography of abdomen revealing distended gallbladder with calculus (red arrow) measuring 3.1 x 2.1 x 2.2 cm impacted in neck of the gallbladder (case 3).

He was started on analgesics, ceftriaxone, doxycycline and his home medication regimen of folic acid and hydroxyurea was resumed. Despite this, he developed worsening generalized abdominal pain localized to RUQ on day 4 of admission. Abdominal ultrasound while positive for a distended gallbladder with sludge and calculi, could not confirm acute cholecystitis so a computed tomography (CT) of the abdomen was done indicating presence of a 3 cm x 2 cm x 2 cm gallstone in neck of gallbladder with moderate to severe gallbladder distention resulting in possible early cholecystitis (Figure 5). He was evaluated by the general surgery team and planned for laparoscopic cholecystectomy. He, however, signed out against medical advice on day 5 of admission despite extensive counseling.

**Figure 6:**
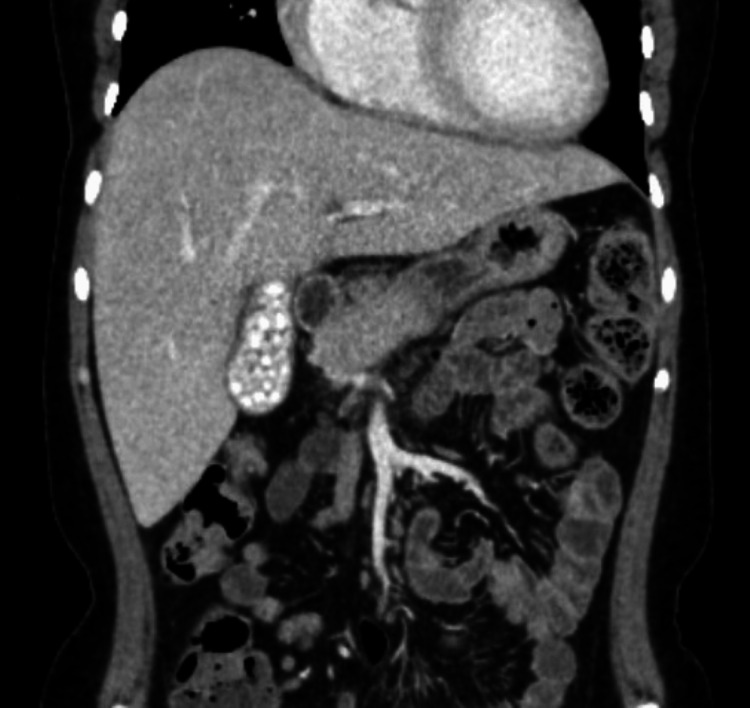
Coronal view of computed tomography of abdomen showing innumerable tiny calcified gallstones filling the entire gallbladder lumen (case 4).

Case 4 involves a 28-year-old woman with SCD and multiple previous admissions for acute chest syndrome who presented with a two-week long history of intermittent epigastric and upper abdominal pain worsening over the last day. Initial lab work revealed Hgb of 8.9g/dL, WBC of 10.7 X 10^3^/uL, reticulocyte count 9.21%, total bilirubin 3.2 mg/dl, and LDH 393 U/L. Abdominal sonogram was indeterminate for cholecystitis but CT of the abdomen confirmed innumerable tiny, calcified gallstones filling the entire gallbladder lumen (Figure 6).

**Figure 7:**
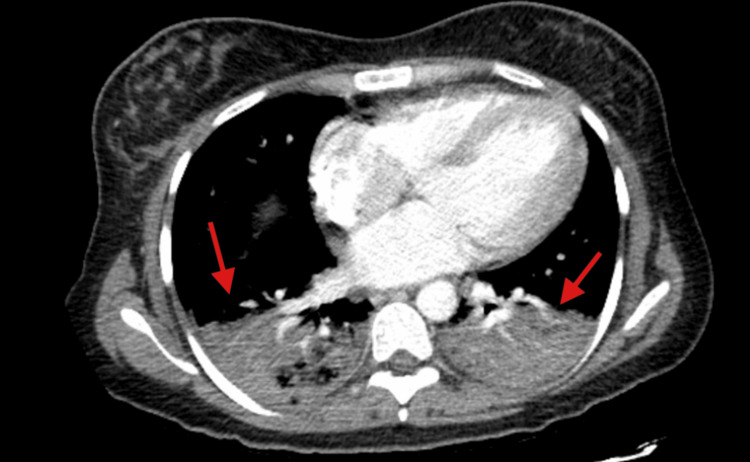
Computed tomography angiogram of chest in case 4 which was negative for pulmonary embolism but positive for bilateral lower zone consolidations (red arrows).

She was started on intravenous fluids, analgesia and then underwent laparoscopic cholecystectomy on day 3 of admission. Post procedure, however, she developed shortness of breath requiring supplemental oxygen, cough, and right shoulder pain. Hemoglobin dropped to 6.9 g/dL and CT angiogram of the chest revealed bilateral lower lung zone consolidations but no pulmonary embolism (Figure 7). CT of the abdomen did not indicate any post-operative hematoma or collection. Due to her clinical condition, she was treated for acute chest syndrome and received exchange transfusion on day 5 of admission. Post-intervention, her symptoms improved, hemoglobin stabilized, and she continued to recover without further complications during the admission.

## Discussion

Hepatobiliary manifestations of SCD occur in 10-40% of sickle cell crisis and include chronic complications such as cholelithiasis, choledocholithiasis, sickle cell cholangiopathy and acute sickle cell hepatic crisis, hepatic cholestasis and hepatic sequestration [2].

As hemolysis of sickled cells occurs, levels of unconjugated bilirubin rise with precipitation of bilirubinate leading to formation of pigmented (typically black) gallstones as opposed to the cholesterol stones seen in the general population. The incidence of cholelithiasis amongst those with HbSS phenotype ranges from 26-58% with incidence in adult patients occurring at the higher rates [3]. Factors which can increase the chance of cholelithiasis in SCD patients include low hemoglobin levels, lower Hgb F levels, higher total serum bilirubin values, and higher reticulocyte counts, all of which are also indicators of higher rates of hemolysis.

Those with cholelithiasis may commonly experience fatty food intolerance with biliary colic type pain presenting as right upper quadrant pain just as our patients did. They may then go on to develop a vaso-occlusive pain crisis, which are commonly precipitated by states of deoxygenation such as hypoxia, dehydration, stress, low temperature, acidosis and infection. Our first case had past presentations for RUQ pain and was found to have cholelithiasis with a dilated CBD of 15 mm without obstruction on MRCP abdomen done previously, but at that time no plan for cholecystectomy was made. Interestingly, in patients with pigmented gallstones, incremental hyperbilirubinemia may be considered a better predictor of choledocholithiasis than is elevated alkaline phosphatase and biliary duct dilatation on imaging, possibly due to the smaller caliber of pigmented stones compared to cholesterol stones that are associated with classical diagnostic findings [4].

Generally, it is recommended that SCD patients who have symptomatic gallstones be referred for cholecystectomy, usually done laparoscopically and requiring a multi-disciplinary approach as such patients must be optimized prior to surgery to ensure favorable outcomes and less complicated post operative courses. However, there is no recognized policy on whether cholecystectomies should be done on SCD patients with asymptomatic gallstone disease [5]. By removing the gallbladder of patients with gallstones, we are getting rid of not just the cause of possible gallstone complications but are also eliminating a differential diagnosis in those SCD patients who may present to us with nonspecific signs such as jaundice with abdominal pain, RUQ pain and fever. Thus, diagnostic dilemmas are avoided and a focus on medical management of the other hepatobiliary complications can be made. By performing elective cholecystectomies in these patients before complications arise, one can also avoid the heavy burden of peri/post operative complications associated with emergency procedures [6], estimated to be ~ 30 % in the SCD population [7]. Perioperative management with intravenous fluids, antibiotics, and blood transfusions with a target Hgb of 10 g/dL, were recommended for better outcomes in some studies, and exchange transfusions were utilized in SCD patients who were known to have frequent SCD-related crisis or other complications [5,7].

It is estimated that 70% of those with SCD will develop gallstones by their third decade of life, which brings us to the need for screening of such patients for cholelithiasis [8]. The use of ultrasound to detect gallstones is accepted worldwide as the modality is readily available and very reliable [9]. It is less costly than other imaging modalities and should be performed in adolescents and all adults with some research advocating for as early as 11 years of age to begin screening and providing patient education [10].

## Conclusions

This study highlights the clinical benefits of screening for gallstones and counseling SCD patients with gallstones on elective cholecystectomy in order to prevent the development of downstream biliary complications that are likely to occur. This consideration stands to garner even more support in its application in younger patients as it can serve to prevent episodes of biliary colic, cholecystitis and potentially choledocholithiasis, thus reducing future hospitalization rates and dramatically improving the quality of life of SCD patients. Future research and guidelines should focus on establishing protocols for screening and management of gallstone-related hepatobiliary complications in this population.
